# ZLN005 Alleviates In Vivo and In Vitro Renal Fibrosis via PGC-1α-Mediated Mitochondrial Homeostasis

**DOI:** 10.3390/ph15040434

**Published:** 2022-03-31

**Authors:** Pengfei Zhu, Haijian Ma, Shichao Cui, Xiqiao Zhou, Weilong Xu, Jiangyi Yu, Jingya Li

**Affiliations:** 1 The First Clinical Medical School, Nanjing University of Chinese Medicine, Nanjing 210000, China; 20193026@njucm.edu.cn; 2 State Key Laboratory of Drug Research, The National Center for Drug Screening, Shanghai Institute of Materia Medica, Chinese Academy of Sciences, Shanghai 201203, China; mahaijian@hotmail.com (H.M.); sccui@simm.ac.cn (S.C.); 3 Department of Endocrinology, Jiangsu Province Hospital of Chinese Medicine, Affiliated Hospital of Nanjing University of Chinese Medicine, Nanjing 210000, China; zhouxiqiao@njucm.edu.cn (X.Z.); 13814000820@163.com (W.X.); 4 School of Pharmaceutical Science and Technology, Hangzhou Institute for Advanced Study, University of Chinese Academy of Sciences, Hangzhou 310000, China

**Keywords:** ZLN005, PGC-1α, mitochondrial homeostasis, UUO

## Abstract

Currently, chronic kidney disease (CKD) is one of the most common diseases; it is also a serious threat to human health due to its high mortality, and its treatment is still a major clinical challenge. Mitochondrial dyshomeostasis plays an important role in the development of CKD. ZLN005 is a novel peroxisome-proliferator-activated receptor-γ coactivator-1α (PGC-1α) activator from our laboratory. To explore whether ZLN005 can protect against CKD in vivo and in vitro, a unilateral ureteral obstruction (UUO) model and TGF-β1-treated renal tubular epithelial cells (TECs), respectively, were used in this study. We found that ZLN005-administrated UUO mice showed less kidney damages than control mice, as indicated by the reduced expression of fibrotic biomarkers in the kidney of UUO mice. ZLN005 treatment also alleviated the TGF-β1-induced fibrotic phenotype and lipid accumulation in TECs. Our study demonstrated ZLN005 treatment improved mitochondrial homeostasis at least partially via the activation of PGC-1α, thus maintaining mitochondria function and energy homeostasis. In summary, ZLN005 treatment ameliorates UUO-induced renal fibrosis, providing conceptional support for mitochondria-targeting therapies for chronic kidney disease.

## 1. Introduction

Tissue fibrosis is a pathological condition that significantly influences organ function with high morbidity and mortality, which are serious threats to human health and property. In the kidney, fibrosis indicates the progression of chronic kidney failure, which is major pathology of chronic kidney disease (CKD) [1,2]. CKD is typically defined as a reduction in kidney function, an estimated glomerular filtration rate (eGFR) of less than 60 mL/min per 1.73 m^2^, or an increase in markers of kidney damage that are present for at least 3 months [3]. CKD contributes to a growing global burden, which is predicted to be the fifth leading cause of death worldwide and is becoming a major biomedical challenge [4]. 

For a long time, researchers of renal fibrosis have focused on fibroblasts and myofibroblasts. However, with further research, the role of tubular epithelial cells (TECs) in renal fibrosis has been continuously highlighted. TECs are the initiators of and participants in renal fibrosis, especially in renal interstitial fibrosis [5]. TECs, particularly proximal tubular epithelial cells, lack hexokinase, resulting in their weaker ability to use glucose for energy. As the most energy-intensive organs, proximal TECs provide energy mainly through fatty acid oxidation (FAO) [6]. A genome-wide transcriptome study of normal and fibrotic kidneys of humans and mice showed that the biopsy specimens with renal interstitial fibrosis present a reduced expression of key enzymes and regulators of FAO and higher lipid accumulation [7]. In another study, researchers demonstrated that overexpression of CPT1A, one of the key enzymes of FAO, significantly improved renal fibrosis in different mouse models [8]. These studies indicate that restoring FAO can attenuate renal fibrosis.

Peroxisome-proliferator-activated receptor-γ coactivator-1 alpha (PGC-1α) is the key regulator of mitochondrial biogenesis, playing a vital role in the maintenance of mitochondrial homeostasis, binding with nuclear respiratory factor 1 (NRF1); nuclear factor erythroid 2-related factor 2 (NRF2) mediates activation of downstream gene transcription and translation, including most of FAO-related genes and mitochondrial DNA (mtDNA) regulator genes [9]. Thus, activating PGC-1α will accelerate mitochondria restoration and promote FAO, thereby expediting renal interstitial fibrosis repair. A previous study demonstrated that restoration of PGC-1α activity could be a potential therapeutic strategy against diabetic kidney disease [10]. However, no study to date has been reported to pharmacologically elevate the PGC-1α levels and evaluate the subsequent effects in a unilateral ureteral obstruction (UUO)-induced renal fibrosis model in mice, which simulates human chronic obstructive kidney disease research [11].

The PGC-1α activator ZLN005, a compound originally developed in our laboratory, has been demonstrated to improve acute kidney injury in different mice models by improving mitochondrial dysfunction [12,13,14]. In this study, we aimed to investigate ZLN005’s protective role in promoting mitochondrial homeostasis to improve the UUO-induced renal fibrosis in mice.

## 2. Results

### 2.1. ZLN005 Treatment Alleviates UUO-Induced Renal Damage and Fibrosis

**Figure 1:**
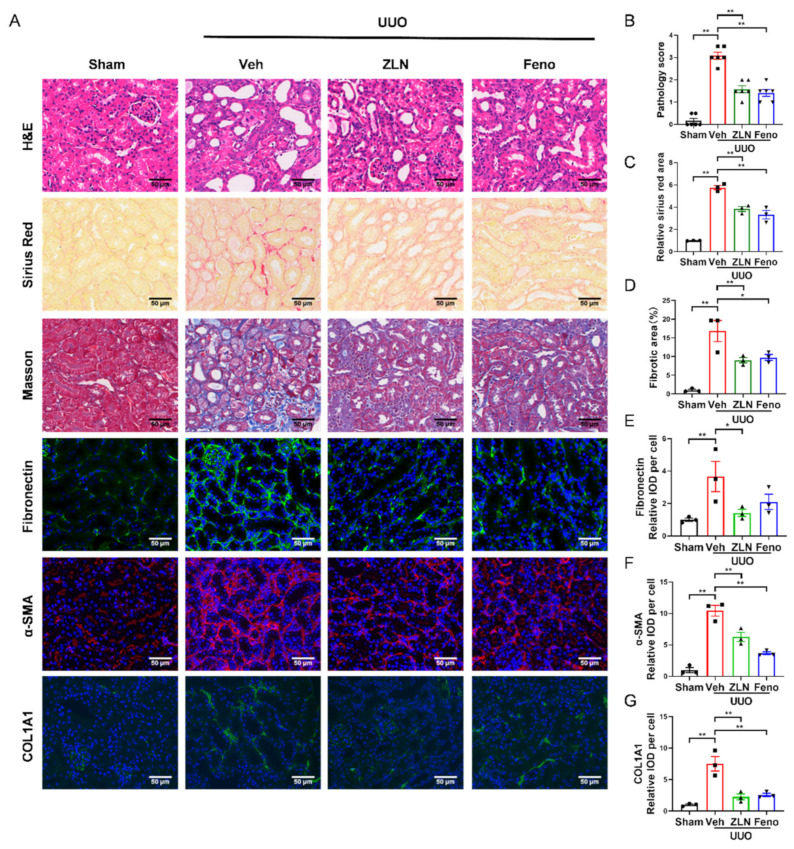
ZLN005 alleviates UUO-induced renal damage and fibrosis. (**A**) Histological changes were assessed by hematoxylin and eosin (H&E) staining. Fibrosis was assessed by Sirius Red and Masson′s trichrome staining. Fibrosis biomarkers Fibronectin, α-SMA and COL1A1 and immunohistochemical staining were used to evaluate fibrosis. Bar = 50 μm. (**B**) The pathologic score of tubular damage. Data are expressed as the mean ± SEM. (*n* = 6). ** *p* < 0.01. (**C**) Relative area of Sirius Red staining was quantified. Data are expressed as the mean ± SEM. (*n* = 3). ** *p* < 0.01. (**D**) The percent of positive area indicated by Masson’s trichrome staining was quantified. Data are expressed as the mean ± SEM. (*n* = 3). * *p* < 0.05, ** *p* < 0.01. Quantification of Fibronectin (**E**), α-SMA (**F**) and COL1A1 (**G**) fluorescence intensity. Data are expressed as the mean ± SEM. (*n* = 3). * *p* < 0.05, ** *p* < 0.01. ZLN005 is abbreviated to ZLN and fenofibrate is abbreviated to Feno.

To investigate the effect of ZLN005 on renal fibrosis, a UUO mouse model was established. After one week, H&E, Masson’s trichrome and Sirius Red staining were used to evaluate renal damage and fibrosis. We found that ZLN005 treatment significantly improved UUO-induced renal proximal tubular histopathological alterations and pathological scores, while the obstructed kidneys from UUO mice without treatment exhibited severe structural disorders, characterized by tubular dilation and atrophy, inflammatory cell infiltration and ECM deposition (Figure 1A,B). Masson’s trichrome and Sirius Red staining showed that ZLN005 significantly decreased renal fibrosis in tubulars after UUO (Figure 1A,C,D). Peroxisome-proliferator-activated receptor-a (PPARα) acts together with PGC-1α to regulate FAO [7,15], and previous studies revealed that the PPARα agonist fenofibrate could effectively improve renal fibrosis [16,17]. Thus, we used fenofibrate as the positive control. The effect of ZLN005 treatment on renal damage and fibrosis was as powerful as that of fenofibrate. To further evaluate the effect of ZLN005 on UUO-induced renal fibrosis, we analyzed the expression of several fibrosis biomarkers, including Fibronectin, α-SMA and COL1A1. Immunofluorescence analysis showed that the vehicle treatment group expressed much higher levels of Fibronectin (Figure 1A,E), α-SMA (Figure 1A,F) and COL1A1 (Figure 1A,G) compared to the sham control. ZLN005 and fenofibrate treatment attenuated the expression of all the fibrosis biomarkers examined (Figure 1A,E–G). Furthermore, quantitative RT-PCR indicated the mRNA expression of *α-SMA*, *Col1a1*, *Col3a1* and *Col4a1* was reduced in ZLN005 and fenofibrate-treated mice (Figure S1A–D).

### 2.2. ZLN005 Treatment Alleviates UUO-Induced Renal Inflammatory Responses

**Figure 2:**
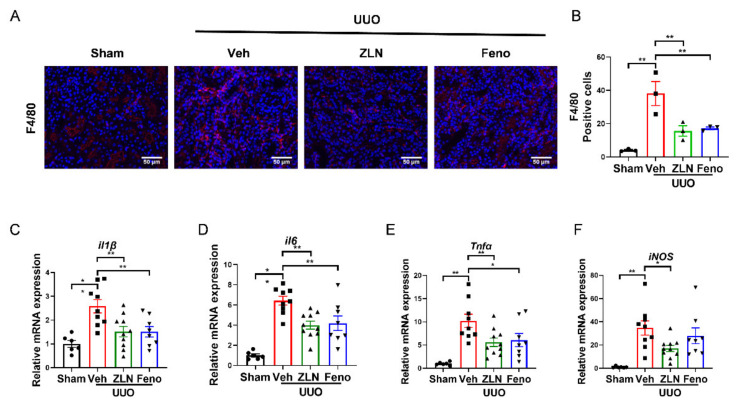
ZLN005 treatment inhibits UUO-induced renal inflammation. (**A**) Representative Immunohistofluorescence for F4/80. Bar = 50 μm. (**B**) Representative of statistical quantitative analysis of F4/80 positive cells. Data are expressed as the mean ± SEM. (*n* = 3). ** *p* < 0.01. The mRNA level of *il1β* (**C**), *il6* (**D**), *Tnfα* (**E**), *iNos* (**F**) were measured for evaluation the kidney inflammation. Data are expressed as the mean ± SEM. (*n* = 6~10). * *p* < 0.05, ** *p* < 0.01. ZLN005 is abbreviated to ZLN and fenofibrate is abbreviated to Feno.

Inflammation is one of the key factors in the occurrence and progression of fibrosis [18]. The obstructed kidney is characterized by inflammatory cell infiltration and increased inflammatory cytokine production [19]. The immunofluorescence of F4/80 showed the macrophage infiltration. It was obvious that the vehicle treatment group had significantly more positive cells than the sham group. The ZLN005 and fenofibrate-treated group presented a significantly reduced number of positive-stained cells, indicating macrophage infiltration was effectually inhibited (Figure 2A,B). The results for the expression of several inflammatory cytokines as measured by quantitative RT-PCR display that ZLN005 and fenofibrate treatment effectively reduced the mRNA expression of *il-1β*, *il6*, *Tnfα* and *iNos* mRNA (Figure 2C–F).

### 2.3. ZLN005 Improves Mitochondrial Homeostasis and FAO in UUO-Induced Renal Fibrosis

**Figure 3:**
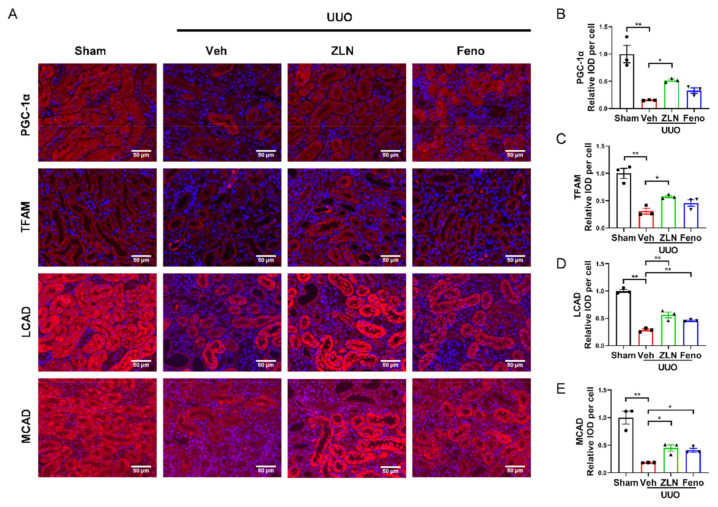
ZLN005 improves mitochondrial homeostasis and FAO in UUO-induced renal fibrosis. (**A**) Immunohistochemical staining of PGC-1α, TFAM, LCAD and MCAD was performed to examine kidney mitochondrial homeostasis. Bar = 50 μm. Quantification of PGC-1α (**B**), TFAM (**C**), LCAD (**D**) and MCAD (**E**) fluorescence intensity. Data are expressed as the mean ± SEM. (*n* = 3). * *p* < 0.05, ** *p* < 0.01. ZLN005 is abbreviated to ZLN and fenofibrate is abbreviated to Feno.

For further determination of the effect of ZLN005 on mitochondrial biogenesis in vivo, the protein expression of PGC-1α and TFAM was detected by immunofluorescence. The results show that the fluorescence intensities were significantly increased in ZLN005-treated group compared with the vehicle treatment group (Figure 3A–C). Protein expression of PGC-1α and TFAM in the fenofibrate treatment group was increased, but had no significant differences. The levels of mRNA expression of *Ppargc1α* and *Tfam* showed nearly the same trend as the protein level (Figure S1E,F). It is widely recognized that renal interstitial fibrosis is associated with low expression of key enzymes and regulators of FAO and high intracellular lipid deposition [7]. The FAO key enzymes, such as LCAD and MCAD, detected in this study by immunofluorescence confirmed ZLN005 treatment drastically increased LCAD and MCAD expression in the kidney after UUO surgery, which indicates ZLN005 has the capacity to promote FAO (Figure 3A,D,E). The mRNA expression of *Cpt1α*, *Lcad* and *Mcad* suggested similar results as well (Figure S1G–I). It is worth mentioning that mRNA levels of some fatty acid synthetases were increased after UUO, and ZLN005 treatment could reduce the expression of *ACC*, *Fas*, *Srebp1c* and *Dgat1* (Figure S1J–M). The positive control fenofibrate treatment showed similar results as the ZLN005-treated group (Figure 3A,D,E and Figure S1G–M).

### 2.4. ZLN005 Alleviates the Changes of TGF-β1-Induced Renal Tubular Epithelial Cells to Fibrotic Phenotype through PGC-1α

**Figure 4:**
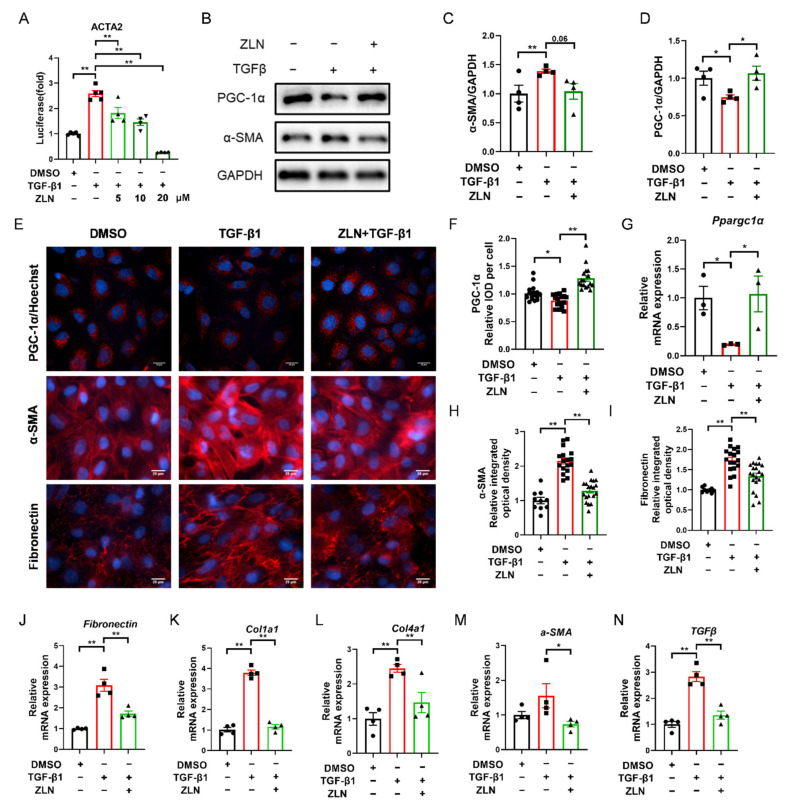
ZLN005 alleviates TGF-β1-induced renal tubular epithelial cells fibrosis through PGC-1α. (**A**) Inhibitory effects of ZLN005 in ACTA2 luciferase assay. Data are expressed as the mean ± SEM. (*n* = 4~5). ** *p* < 0.01. Expression of PGC-1α and α-SMA in HKC cells lysates was detected by Western blot (**B**) and quantified by densitometry (**C**,**D**). Data are expressed as the mean ± SEM. (*n* = 4). * *p* < 0.05. (**E**) Representative images of immunocytofluorescence staining of PGC-1α, α-SMA and Fibronectin. Bar = 20 μm. Quantification of PGC-1α (**F**), α-SMA (**H**) and Fibronectin (**I**) fluorescence intensity. Data are expressed as the mean ± SEM. (PGC-1α: *n* = 17; α-SMA and Fibronectin: DMSO: *n* = 9, other group: *n* = 17~20,). ** *p* < 0.01. The mRNA levels of *Ppargc1α* (**G**), *Fibronectin* (**J**), *Col1a1* (**K**), *Col1a4* (**L**), *α-SMA* (**M**) and *TGFβ* (**N**) were measured for evaluation of TECs fibrosis. Data are expressed as the mean ± SEM. (*n* = 4). * *p* < 0.05, ** *p* < 0.01. ZLN005 is abbreviated to ZLN.

TGF-β1 is a major pro-fibrogenic cytokine [20]. In order to determine whether ZLN005 could improve TGF-β1-induced TECs fibrosis, a fluorescent reporter plasmid of α-SMA was transfected into NRK-52E cells. The luciferase activity was increased by TGF-β1 and reduced by ZLN005 after multiple doses (Figure 4A). The intermediate dose (ZLN005 10 μM) of the effective dose was selected for subsequent cell experiments. Immunoblotting showed that an increase in PGC-1α after ZLN005 treatment could improve the change of the cells to a fibrosis phenotype as indicated by decreased expression of α-SMA (Figure 4B–D). Immunofluorescence staining of PGC-1α, α-SMA and Fibronectin showed the same result as well (Figure 4E,F,H,I). ZLN005 treatment upregulated mRNA expression of *Ppargc1α* and reduced mRNA expression of fibrosis biomarkers for *Fibronectin*, *Col1a1*, *Col4a1*, *α-SMA* and *TGFβ* at the same time (Figure 4G,J–N).

### 2.5. ZLN005 Inhibits Lipid Accumulation by Improving Mitochondrial Homeostasis

**Figure 5:**
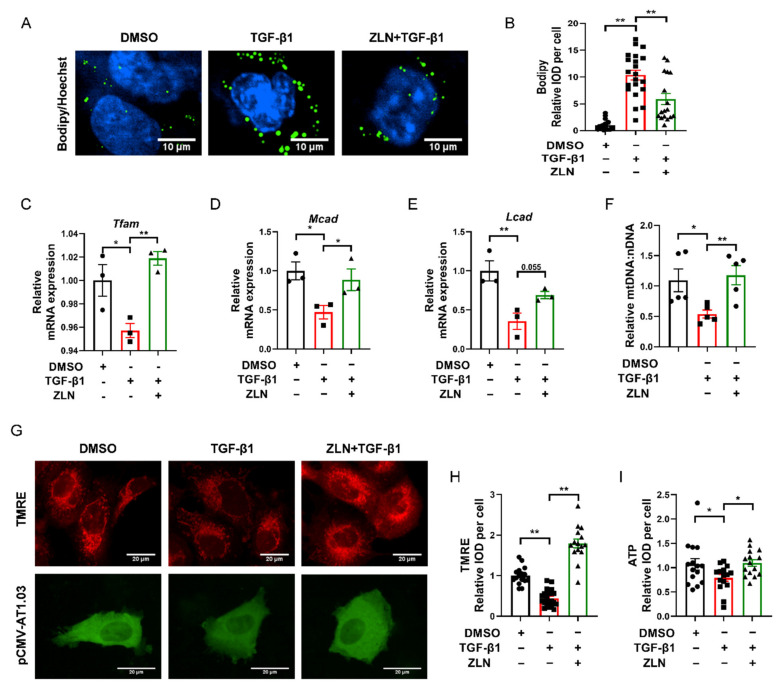
ZLN005 inhibits lipid accumulation by improving mitochondrial homeostasis. (**A**) Representative images of lipid droplets detected with Bodipy in HKC cells. Bar = 10 μm. (**B**) Quantification of lipid droplets fluorescence intensity in each cell. Data are expressed as the mean ± SEM. (*n* = 17). ** *p* < 0.01. Level of *Tfam* (**C**), *Mcad* (**D**) and *Lcad* (**E**) mRNA were detected by quantitative RT-PCR. Data are expressed as the mean ± SEM. (*n* = 3). * *p* < 0.05, ** *p* < 0.01. (**F**) Relative mitochondrial DNA copy number (mtDNA: nDNA). Data are expressed as the mean ± SEM. (*n* = 5~6). * *p* < 0.05. (**G**) Representative images of mitochondrial membrane potential detected by TMRE dye; ATP level revealed by tool plasmid pCMV-AT1.03. Bar = 20 μm. (**H**) Quantification of TMRE fluorescence intensity in each cell. Data are expressed as the mean ± SEM. (*n* = 15~20). ** *p* < 0.01. (**I**) Quantification of pCMV-AT1.03 fluorescence intensity in each cell. Data are expressed as the mean ± SEM. (*n* = 15). * *p* < 0.05; ** *p* < 0.01. ZLN005 is abbreviated to ZLN.

Lipid accumulation is closely related to the development of fibrosis in TECs. It has been suggested that excessive accumulation of triglycerides can induce cellular lipotoxicity, which contributed to the development of renal fibrosis [21,22,23]. Bodipy was used to test lipid droplets’ accumulation in TGF-β1-induced HKC cells fibrosis. We noticed that lipid droplets were remarkably increased in TGF-β1 treatment cells, while ZLN005-treated cells presented a dramatic improvement in lipid droplet accumulation (Figure 5A,B). ZLN005-alone treatment on HKC cells for 48 h could significantly increased mRNA expression of *Tfam*, *Mcad* and *Lcad* (Figure S2A–D). Likewise, the mRNA level of these proteins was reduced in TGF-β1-treated cells, which was significant reversed in the cells treated with ZLN005 (Figure 5C–E). In order to verify whether ZLN005 could increase the mitochondrial quality, we used JC-1 and TMRE to test the mitochondrial membrane potential. We found ZLN005-treated cells, after 2 h, showed a more significant increase in mitochondrial depolarization than the DMSO group (Figure S2E,F). Meanwhile, the mitochondrial membrane potential was drastically decreased by TGF-β1 and markedly reversed by ZLN005 treatment (Figure 5G,H). As is known to all, the mitochondrial membrane potential is vital for maintaining the respiratory chain to generate ATP. Then, we tested the effects of ZLN005 on cytosolic ATP production with the tool plasmid pCMV-AT1.03. As predicted, the ATP level of TGF-β1-treated cells was reduced compared to DMSO-treated control cells, while ZLN005 treatment partially recovered the capacity of cells to produce energy (Figure 5G,I). We also measured the ratio of mtDNA:nDNA. ZLN005 treatment significantly upregulated the mtDNA copy number that was reduced due to TGF-β1 treatment (Figure 5F).

Collectively, ZLN005 could regulate mitochondrial homeostasis and the expression of key enzymes of FAO by upregulating PGC-1α, thus improving renal fibrosis.

### 2.6. ZLN005 Improvement of TGF-β1-Induced TECs Fibrosis Partially Depends on PGC-1α

**Figure 6:**
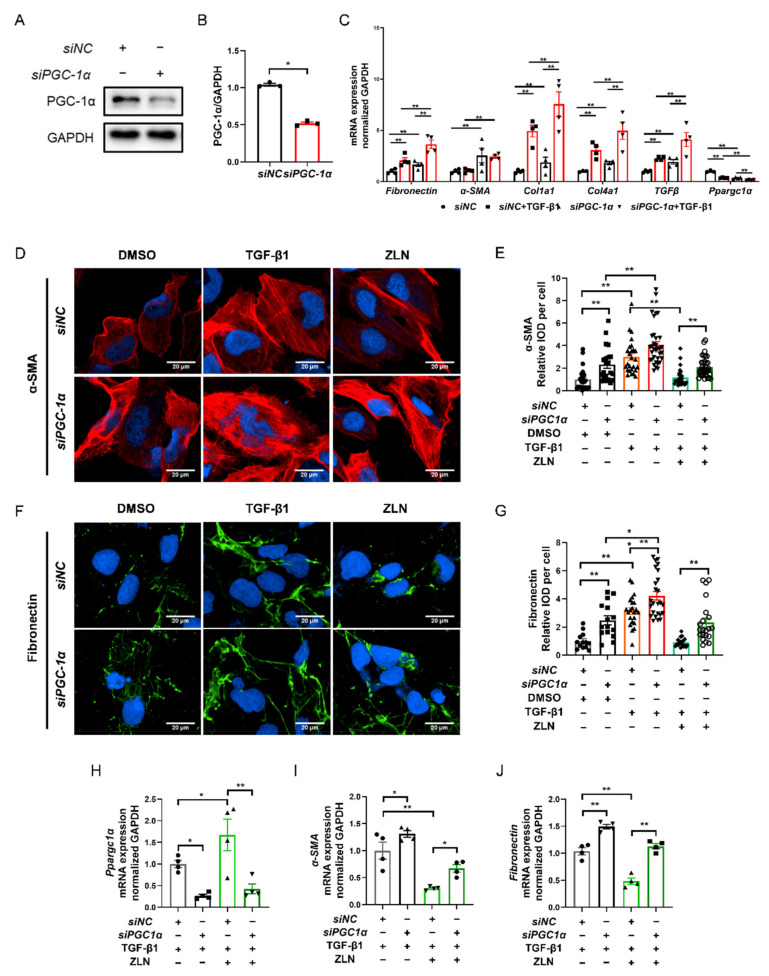
ZLN005 improves TGF-β1-induced renal tubular epithelial cell fibrosis partially depends on PGC-1α. (**A**) Western blotting was performed to determine expression of PGC-1α protein in siRNA-transfected HKC cells. (**B**) Quantitative analysis of PGC-1α protein levels in control siNC- or PGC-1α-siRNA-transfected HKC cells. Data are expressed as the mean ± SEM. (*n* = 3). ** *p* < 0.01. (**C**) The mRNA level of *Fibronectin*, *α-SMA*, *Col1a1*, *Col1a4*, *TGFβ* and *Ppargc1α* were measured in siNC- or PGC-1α-siRNA-transfected HKC cells after the corresponding α treatments. Data are expressed as the mean ± SEM. (*n* = 3~4). ** *p* < 0.01. Representative images of immunocytofluorescence staining of α-SMA (**D**) and Fibronectin (**F**) in siNC- or PGC-1α-siRNA-transfected HKC cells after the corresponding α treatments. Bar = 20 μm. Quantification of α-SMA (**E**) and Fibronectin (**G**) fluorescence intensity in each cell. Data are expressed as the mean ± SEM. (α-SMA: *n* = 21~35, Fibronectin: *n* = 15~25). * *p* < 0.05, ** *p* < 0.01. Level of *Ppargc1α* (**H**), *α-SMA* (**I**) and *Fibronectin* (**J**) mRNA were detected by quantitative RT-PCR in HKC cells after the corresponding α treatments. Data are expressed as the mean ± SEM. (*n* = 4). * *p* < 0.05, ** *p* < 0.01. ZLN005 is abbreviated to ZLN.

As ZLN005 is a recognized PGC-1α activator, it is interesting to understand that the effect on fibrosis is dependent on PGC-1α. We then knocked down PGC-1α of HKC cells with specific siRNA (Figure 6A,B). The results show that PGC-1α knockdown significantly increased the transcriptional level of fibrosis biomarkers such as *Fibronectin*, *Col1a1*, *Col4a1* and *TGFβ*, and the level was further significantly increased by TGF-β1 treatment (Figure 6C), indicating that PGC-1α deficiency could result in progression of fibrosis. Meanwhile, the protective role of ZLN005 on renal fibrosis was impaired due to PGC-1α knock down, as shown by immunofluorescence staining of α-SMA and Fibronectin (Figure 6D–G). Additionally, the result for the mRNA level of *Ppargc1α*, *Fibronectin* and *α-SMA* showed the same trend (Figure 6H–J). Finally, we found that the treatment of ZLN005 on TECs fibrosis may at least partially dependent on PGC-1α.

## 3. Discussion

Although myofibroblasts are initial factors in the development of renal fibrosis, renal proximal tubular cells play a central role in chronic kidney disease [24,25,26,27]. The kidney is one of the most energy-demanding organs in the human body and is only second to the heart in mitochondrial abundance, especially in renal tubular cells [28,29]. Fatty acids have been identified as the major energy source in tubular cells, and FAO is the main way in which ATP is produced [30]. These all count on the proper functioning of mitochondria. In previous studies, mitochondrial dysfunction has been shown to be a key factor in kidney disease [31,32]. Due to the heterogeneity of kidney disease, mitochondria-targeting therapies, acting upstream of damaged cells, could be more advantageous than targeting downstream [33,34,35]. Improvement of mitochondrial function enhances mitochondria homeostasis, including mitochondrial biogenesis, mitochondrial fusion, fission and mitophagy. In a previous study, we proved that regulating mitophagy and promoting FAO protected ischemia/reperfusion-induced acute kidney injury [36]. In the present study, we investigated the effect of pharmacological enhancement of PGC-1α on CKD. 

It has been reported that intracellular lipid accumulation can be observed in patients and mice with tubulointerstitial fibrosis [7]. In proximal tubular cells, fatty acid can be taken up by membrane fatty acid transport proteins, such as CD36 and FATP2.33. However, mice overexpressing CD36 in tubules did not develop kidney fibrosis despite increased lipid levels in tubular epithelial cells [37]. In contrast, inhibition of FAO exacerbated the fibrotic phenotype in tubule epithelial cells, increased cell death and triggered intracellular lipid accumulation in vitro [37]. Furthermore, pharmacological enhancement of FAO protected mice from kidney fibrosis [37]. Miguel et al. revealed that enhanced fatty acid oxidation by modulating CPT1A protected against kidney fibrosis [8]. This evidence suggests the imbalance in fatty acid uptake and utilization contributes to the accumulation of fatty acids in the tubular cells, thereby leading to the enhanced lipid accumulation and mitochondrial dysfunction. PGC-1α, a key transcriptional regulator of fatty acid uptake and oxidation, has been proven to be significantly decreased in patients with CKD and kidney fibrosis [8]. In a previous study, Smad3, which is a downstream effector of TGF-β [38], can directly suppress expression of PGC-1α [7,39], Therefore, fibrosis induced by TGF-β could be improved by enhancing PGC-1α. PPARα acts together with PGC-1α to regulate FAO and improves fibrosis by regulating renal lipolysis through the upregulation of expression levels of several enzymes involved in lipolysis [15,40]. Fenofibrate is a well-known PPARα agonist which has been reported to significantly improve renal fibrosis [16,17]. In this study, fenofibrate could significantly improve the protein level of key enzymes of FAO, which indicates fenofibrate could improve renal fibrosis through FAO. ZLN005, the first small-molecule PGC-1α activator, has been proved to be protected against multiple diseases by activating PGC-1α. ZLN005 significantly improved the renal fibrosis phenotype and reduced TGF-β1-induced lipid accumulation in TECs by enhancing the levels of key enzymes for FAO, suggesting that ZLN005 improved mitochondrial homeostasis by regulating PGC-1α co-transcription target genes’ expression in the kidney. ZLN005 may also improve fibrosis by affecting the binding of Smad3 to an intronic area on the *PPARGC1α* gene, which requires further research. 

As the key regulator of mitochondrial biogenesis, PGC-1α is important in the recovery of renal injury [41]. It has been reported that specific overexpression of PGC-1α in renal tubular cells reduced renal fibrosis by restoring mitochondrial content in folic-acid-induced fibrosis and fibrosis in Notch transgenic mice [7,42]. PGC-1α signaling also up-regulates mitochondria function by activating mitochondrial transcription factor A (TFAM), which in turn regulates mitochondrial DNA transcription. In the present study, ZLN005-treated mice showed a higher TFAM level in the UUO model, which was more significant than that obtained via fenofibrate treatment. Furthermore, the mtDNA copy number also showed a higher level in vitro after TGF-β treatment. The results of TMRE staining and ATP levels also showed ZLN005 significantly increased the mitochondrial membrane potential and mitochondrial function in vitro, which suggests ZLN005 could improve mitochondrial homeostasis. After a knockdown of PGC-1α expression by siRNA, the efficacy of ZLN005 was blocked in the fibrosis model in vitro, which suggests ZLN005 protection against kidney fibrosis may be partially dependent on PGC-1α. 

In summary, this study provided substantial evidence for the efficacy of ZLN005 in in vitro and in vivo models of renal fibrosis. ZLN005 improves mitochondrial homeostasis, helping renal tubular cells against fibrosis, which suggests ZLN005 might be a potential therapeutic compound for renal fibrosis.

## 4. Materials and Methods

### 4.1. Antibodies and Reagents

Antibody against PGC-1α (NBP1-04676) was obtained from Novus Biologicals (Centennial, CO, USA). TFAM (22586-1-AP), MCAD (55210-1-AP) and LCAD (17526-1-AP) antibodies were purchased from Proteintech (Wuhan, Hubei, China). Antibodies to fibronectin (ab2413) were purchased from Abcam (Cambridge, CB, UK). Antibody to α-SMA (ARG66381) was purchased from ArigoBio (Hsinchu, Taiwan, China). Antibody to COL1A1 (SC-293182) was purchased from Santa Cruz (Santa Cruz, CA, USA). Antibody to GAPDH (14C10) was obtained from Cell Signaling Technology (Farmingdale, NY, USA). Compound ZLN005 (HY-17538) and fenofibrate (HY-17356) were purchased from MedChemExpress (Shanghai, China). TGF-β1 (10804-HNAC) was purchased from Sino Biological (Beijing, China). Hoechst 33342 (C1022), JC-1 kit (C2006) and TMRE kit (C2001S) were purchased from Beyotime Biotechnology (Shanghai, China). Bodipy (D3922) was purchased from Thermo Fisher Scientific (Waltham, MA, USA).

### 4.2. Animals

Male C57 BL/6J mice were purchased from Vital River Laboratory Animal Corporation (Beijing, China) and housed in a pathogen-free animal facility under a 12 h light–dark pattern with free access to food and water. All animal experiments were conducted by a protocol approved by the Institutional Animal Care and Use Committee of Shanghai Institute of Materia Medica, Chinese Academy of Sciences.

### 4.3. UUO Models and Treatment

Mice were randomly assigned to four groups: (1) sham-operated mice (sham); (2) UUO mice with vehicle (Veh); (3) UUO mice treated with ZLN005 (ZLN); and (4) UUO mice treated with fenofibrate (Feno). To establish UUO model, mice were anesthetized with pentobarbital (75 mg/kg, i.p.). Unilateral abdominal incision was performed to expose the left ureter and ligated with 4-0 silk at two points, and then the ureter was cut between the 2 ligation points. The sham-operated group did not undergo ligation. For in vivo experiments, ZLN005 (40 mg/kg) and fenofibrate (100 mg/kg) were dissolved in 0.5% CMC-Na, and mice were pretreated with ZLN005 (40 mg/kg, i.g.) and fenofibrate (100 mg/kg, i.g.) for one day. UUO surgery was performed 1 h after day 2 of oral administration. One week later, mice were treated with ZLN005 and fenofibrate daily. The vehicle group received 0.5% CMC-Na alone as vehicle. The mice were sacrificed, and the left kidneys were collected at 7 days after UUO surgery. Sections were stained with H&E, Masson’s trichrome and Sirius Red to evaluate histological change and fibrosis. Ten non-repeating fields were randomly selected. Tubular lesions were scored from 0 to 5 [31], as follows: 0: normal; 1: mild (<25% of the cortex); 2: moderate (25~50%); 3: severe (50~75%); and 4: extensive damage (>75%). The positive areas of Masson’s trichrome staining (blue) and Sirius Red staining (red) were calculated by ImageJ (NIH).

### 4.4. Cell Culture and Treatment

Human renal proximal tubular cell line (HKC) was purchased from BeNa Culture Collection (Beijing, China) and rat renal proximal tubular cell line (NRK-52E) was obtained from Cell Bank/Stem Cell Bank, Chinese Academy of Sciences (Shanghai, China). NRK-52E cells were cultured in HG-DMEM medium containing 10% FBS, penicillin and streptomycin. HKC cells were cultured in DMEM/F12 medium with 10% FBS, containing penicillin and streptomycin. All the cells were placed in an incubator at 37 °C with 5% CO_2_, and the medium was replaced every two days. When cells grew to ~80% confluence, they were digested with 0.25% trypsin-0.02% EDTA for passage. The cell treatment followed a previous study [7]. Briefly, at 80% confluence, cells were starved in RPMI1640 containing 0.5% FBS overnight and treated with 50 ng/mL TGF-β1 with or without 10 μM ZLN005 for 48 h.

### 4.5. Immunohistofluorescence Analysis

The protocol of immunohistofluorescence analysis followed that of our previous study [36]. After staining, the sections were scanned by Vectra imaging system (PerkinElmer, Waltham, MA, USA). For quantification, 20–25 fields were randomly selected and calculated by ImageJ (NIH). All the data of immunohistochemical were relative to the number of nuclei.

### 4.6. Plasmids, Short Interfering RNA and Transfection

ACTA2 promoter pGL3-Basic plasmid was purchased from YouBio (Changsha, Hunan, China). The plasmid pCMV-AT1.03 (D2604) was purchased from Beyotime (Shanghai, China). The plasmids were transfected by EZ Trans (AC04L092) from Life-ilab Bio (Shanghai, China). Short interfering RNA (siRNA) oligonucleotides against PGC-1α were obtained, and their negative control siRNAs were synthesized by GenePharma (Shanghai, China). Transient transfections of siRNA were carried out using Lipofectamine RNAimax reagent (13778150) from Thermo Fisher Scientific (Waltham, MA, USA). The sequences of siRNA oligonucleotides were as follows: PGC-1α siRNA, sense: 5′-GATGTGAACGACTTGGATACA-3′ and 5′-GCTTGTTCAGCGGTTCTTTCT-3′, control siRNA, sense: 5′-UUCUCCGAACGUGUCACGU-3′.

### 4.7. Assessment of Mitochondrial Membrane Potential

The membrane-potential-sensitive dye JC-1 kit and TMRE kit were used to measure mitochondrial membrane potential according to the manufacturer’s instructions. The cells were directly examined by Opera Phenix (PerkinElmer, Waltham, MA, USA), and 20–25 fields were randomly selected in each condition.

### 4.8. Quantification of Mitochondrial DNA Content

For this step, we followed the method referred to in our previous research [43]. Total cellular DNA was extracted using the DNA Isolation Mini Kit (DC102-01, Vazyme, Nanjing, Jiangsu, China) according to the manufacturer’s instructions and used for the detection of mtDNA copy number by qPCR with the following reagents: TaqMan Universal PCR master mix and the primers (16S rRNA and 18S rRNA). MtDNA levels were assessed using the mitochondrial genes 16S rRNA; nuclear 18S rRNA served as a loading control.

### 4.9. Immunocytofluorescence and Bodipy Staining

Immunocytofluorescence and Bodipy staining were carried out followed the method of our previous research [43]. In brief, cells were fixed with 4%PFA for 30 min, treated with 0.3% Triton X-100 for 20 min, blocked with 5% BSA for 1 h at room temperature, then incubated with primary antibodies (1:200 dilution) for 1 h and with corresponding secondary antibodies (1:500 dilution) for 1 h at 37 °C. For Bodipy staining, cells were treated with 4%PFA at room temperature for 30 min and incubated with 1 μg/mL Bodipy for 10 min at 37 °C. Subsequently, cells were washed with PBS three times and mounted with Hoechst. The cells were directly examined by Opera Phenix (PerkinElmer, Waltham, MA, USA), and 20–25 fields were randomly selected in each condition.

### 4.10. Quantitative RT-PCR and Western Blot Analysis

For this step, we used the method referred to in our previous research [36]. Total RNA was isolated from kidney or cells using TRIzol reagent (Takara, Kusatsu, Shiga, Japan). One microgram of total RNA was reverse-transcribed using PrimeScript Reverse Transcriptase (Takara). The resulting cDNAs were amplified using 2× SYBR Green qPCR Master Mix (Takara) and a Stratagene Mx3005P instrument (Agilent Technologies, Santa Clara, CA, USA). The data were then normalized to housekeeping gene (GAPDH or Ubiquitin) expression. The primers used for analyses are shown in Supplementary Table S1. Cells lysates were subjected to electrophoresis through SDS-PAGE and transferred to NC membranes and then blocked with 5% nonfat milk. The membranes were incubated with different primary antibodies, followed by incubation with the relative secondary antibodies. Quantification was performed by measuring the band intensities using ImageJ (NIH).

### 4.11. Luciferase Assay

When NRK-52E cells grew to ~70%, ACTA2 promoter pGL3-Basic plasmids were transfected into them by EZ Trans. After 12 h, the cells were starved in RPMI1640 containing 0.5% FBS overnight and treated with 50 ng/mL TGF-β1 with or without different doses of ZLN005 for 48 h, and then luciferase substrates (Promega, Madison, WI, USA) were added to each well. After 30 min, the released luciferin signal was detected using an EnVision microplate reader (Perkin Elmer, Waltham, MA, USA).

### 4.12. Statistical Analysis

The number of samples was 6 for each group in terms of the pathology score, which could accurately show the effect of ZLN005 on the pathological level. Due to the high price of antibodies and remarkable differences in protein levels, we tested 3 samples from each group with immunohistochemical staining. The sample numbers of mRNA levels in in vitro testing by quantitative RT-PCR were 3~4, which was considered to be statistically significant. Upon immunocytofluorescence, Bodipy staining, TMRE staining and other experiments imaged by Opera Phenix, in order to ensure the reliability of results and consistency with our previous research [36], ≥15 random samples of image fields were selected.

The results are presented as the means ± SEM. A non-parametric test, Mann–Whitney test, was used to analyze data for comparisons between two groups, and one-way ANOVA was used to analyze counts between multiple groups. *p*-values < 0.05 were considered to be significant.

## Data Availability

Data is contained within the article.

## Supplementary Materials

The following are available online at https://www.mdpi.com/article/10.3390/ph15040434/s1, Figure S1: mRNA expression of α-SMA (A), Col1a1 (B), Col1a3 (C) and Col1a4 (D) in kidney tissue were examined by Quantitative RT-PCR. Data are expressed as the mean ± SEM. (*n* = 6~9). * *p* < 0.05, ** *p* < 0.01. Level of Ppargc1α (E), Tfam (F), Cpt1α (G), Lcad (H) and Mcad (I) mRNA in kidney tissue were detected by Quantitative RT-PCR. Data are expressed as the mean ± SEM. (*n* = 5~6). * *p* < 0.05, ** *p* < 0.01. The mRNA level of ACC (J), Fas (K), Srebp1c (L) and Dgat1 (M) were measured. Data are expressed as the mean ± SEM. (*n* = 6~8). * *p* < 0.05, ** *p* < 0.01. ZLN005 is abbreviated to ZLN and Feno-fibrate is abbreviated to Feno, Figure S2: ZLN005 alone treatment on HKC cells for 48 h. The mRNA level of Ppargc1α (A), Tfam (B), Mcad(C) and Lcad (D) were measured. Data are expressed as the mean ± SEM. (*n* = 4). * *p* < 0.05, ** *p* < 0.01. One hour treatment of ZLN005 on HKC cells. (E) Representative images of JC-1 staining showing red fluorescence of JC-1 aggregates and green signal of monomers. Bar = 20 μm. (F) Quantification of the ratio of red to green fluorescence. Data are expressed as the mean ± SEM. (*n* = 8~9). ** *p* < 0.01. ZLN005 is abbreviated to ZLN. Table S1: Primer sequences used in the experiments.
