# Supplementary material for: ZLN005 Alleviates In Vivo and In Vitro Renal Fibrosis via PGC-1α-Mediated Mitochondrial Homeostasis
Source: Pharmaceuticals (Basel). 2022 Mar 31;15(4):434. doi: 10.3390/ph15040434 (PMC9025854; doi:10.3390/ph15040434)
Supplement: Supplementary file 1 [file pharmaceuticals-15-00434-s001.zip › pharmaceuticals-1629269-supplementary.pdf]

pharmaceuticals

MDPI

Supplementary Materials: The following supporting information can be downloaded at: www.mdpi.com/xxx/s1.

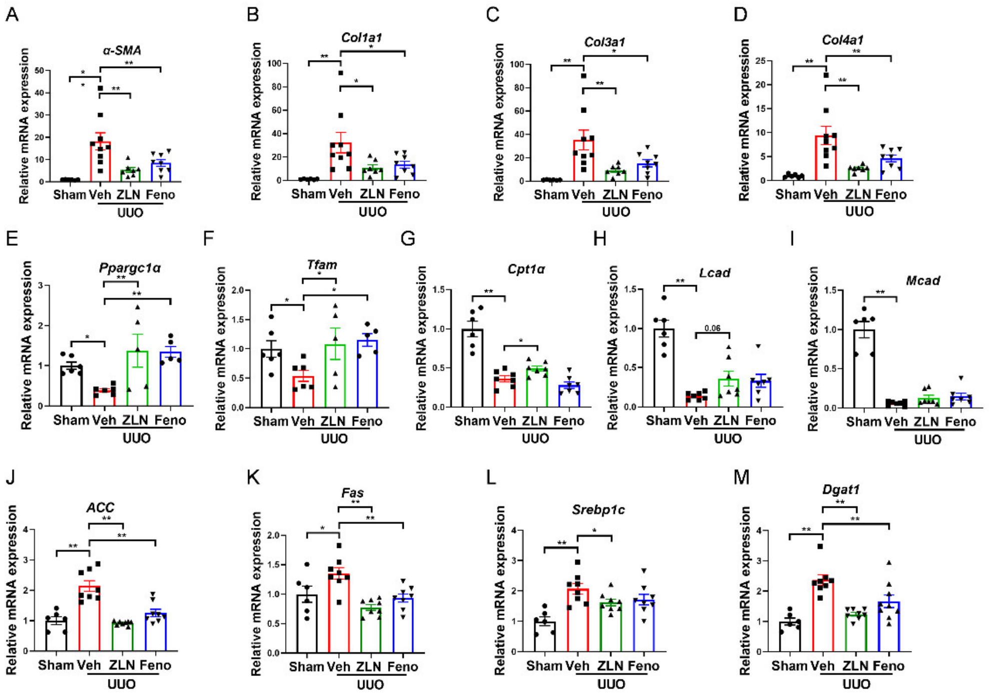

Figure S1. mRNA expression of *α-SMA* (A), *Col1a1* (B), *Col1a3* (C) and *Col1a4* (D) in kidney tissue were examined by Quantitative RT-PCR. Data are expressed as the mean ± SEM. (n=6~9).*P<0.05, **P<0.01. Level of *Ppargc1α* (E), *Tfam* (F), *Cpt1α* (G), *Lcad* (H) and *Mcad* (I) mRNA in kidney tissue were detected by Quantitative RT-PCR. Data are expressed as the mean ± SEM. (n=5~6).*P<0.05, **P<0.01. The mRNA level of *ACC* (J), *Fas* (K), *Srebp1c* (L) and *Dgat1* (M) were measured. Data are expressed as the mean ± SEM. (n=6~8).*P<0.05, **P<0.01. ZLN005 is abbreviated to ZLN and Fenofibrate is abbreviated to Feno.

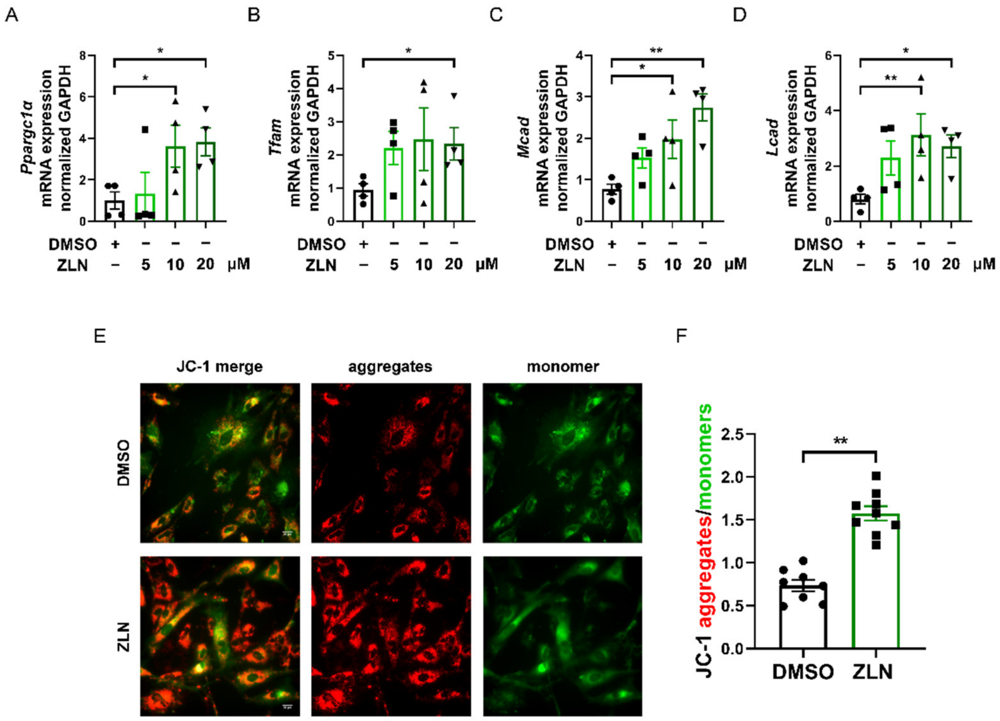

Figure S2. ZLN005 alone treatment on HKC cells for 48 h. The mRNA level of Ppargc1α (A), Tfam (B), Mcad(C) and Lcad (D) were measured. Data are expressed as the mean ± SEM. (n=4).*P<0.05, **P<0.01. One hour treatment of ZLN005 on HKC cells. (E) Representative images of JC-1 staining showing red fluorescence of JC-1 aggregates and green signal of monomers. Bar=20 μm. (F) Quantification of the ratio of red to green fluorescence. Data are expressed as the mean ± SEM. (n=8~9).**P<0.01. ZLN005 is abbreviated to ZLN.

Table 1: Primer sequences used in the experiments.

	**Forward Primer**	**Reverse Primer**
**QPCR primers**		
mGAPDH	TCTCCTGCGACTTCAACA	TGGTCCAGGGTTTCTTACT
mPpargc1α	ACTGAGCTACCCTTGGGATG	TAAGAATTTCGGTGGTGACA
mα-SMA	CTGACAGAGGCACCACTGAA	AGAGGCATAGAGGGACAGCA
mCol1a1	GCTCTTTTTAGATACTGTGGTGAGGAA	GTTTCCACGTCTCACCATTG
mCol3a1	ACAGCTGGTGAACCTGGAAG	ACCAGGAGATCCATCTCGAC
mCol4a1	GACAGCCAGGTTTGACAGGT	GGCAGCTCTCTCCTTTCTGA
mil1β	GCAACTGTTCCTGAACTCAACT	ATCTTTTGGGGTCCGTCAACT
mil6	TAGTCCTTCCTACCCCAATTTCC	TTGGTCCTTAGCCACTCCTTC
miNos	GAGACAGGGAAGTCTGAAGCAC	CCAGCAGTAGTTGCTCCTCTTC
mTnfα	GAAGTTCCCAAATGGCCTCC	TTGTCACTCGAATTTTGAGAAGATG
mTfam	GAGGCAAAGGATGATTCGGCTC	CGAATCCTATCATCTTTAGCAAGC
mACC	TCTACGGCAGCAGTTACACCACAT	TCTCTTCATTACCTCAATCTCAGCATAG
mFas	TGGGTTCTAGCCAGCAGAGT	TACCACCAGAGACCGTTATGC
mSrebp1c	CGACTACATCCGCTTCTTGCAG	CCTCCATAGACACATCTGTGCC

mDgat1	GGAGACCGCGAGTTCTACAG	CTCATGGAAGAAGGCTGAGG
mCpt1α	ATGACGGCTATGGTGTTTCC	TGTCCATCATGGCTTGTCTC
mLcad	TCACCAACCGTGAAGCTCGA	CCAAAAAGAGGCTAATGCCATG
mMcad	AGCTGCTAGTGGAGCACCAAG	TCGCCATTTCTGCGAGC
hGAPDH	GCTCTCTGCTCCTCCTGTTC	ATGGTGTCTGAGCGATGTGG
hPpargc1α	CCCTGTCTGCTCTGTGGACT	GCTCCAAGCTACTGTGGTGA
hα-SMA	ATCACCAACTGGGACGACAT	GGCAACACGAAGCTCATTG
hFibronectin	CCCTGGTGTCACAGAGGCTA	TGTATATTCGGTTCCCGGTTC
hCol1a1	GCGGACTTTGTTGCTGCTTGCAG	ATCTCCGGCTGGGCCCTTTCTT
hCol4a1	CAAGGGCTCGCCGGGTTCTG	CCGGTGTCACCACGACTGCC
hTGFβ	TACCTGAACCCGTGTTGCTCTC	GTTGCTGAGGTATCGCCAGGAA

(Continued on next page)

Continued

	**Forward Primer**	**Reverse Primer**
hTfam	GTGGTTTTCATCTGTCTTGGCAAG	TTCCCTCCAACGCTGGGCAATT
hLcad	GTTTGGACTCCGCCACTGCTTG	GGCTGAACTCTGGCATCCACAT
hMcad	AGAACCTGGAGCAGGCTCTGAT	GGATCTGGATCAGAACGTGCCA
18s rRNA	CTACCACATCCAAGGAAGCA	TTTTCGTCACTACCTCCCCG
16s rRNA	GCCTTCCCCCGTAAATGATA	TTATGCGATTACCGGGCTCT

Ubiquitin	GCCCAGTGTTACCACCAAGAAG	GCTCTTTTTAGATACTGTGGTGAGGAA
